# Fully Integrated PET/MR Imaging for the Assessment of the Relationship Between Functional Connectivity and Glucose Metabolic Rate

**DOI:** 10.3389/fnins.2020.00252

**Published:** 2020-03-25

**Authors:** Lalith Kumar Shiyam Sundar, Shahira Baajour, Thomas Beyer, Rupert Lanzenberger, Tatjana Traub-Weidinger, Ivo Rausch, Ekaterina Pataraia, Andreas Hahn, Lucas Rischka, Marius Hienert, Eva-Maria Klebermass, Otto Muzik

**Affiliations:** ^1^QIMP Team, Center for Medical Physics and Biomedical Engineering, Medical University of Vienna, Vienna, Austria; ^2^Department of Psychiatry and Behavioral Neurosciences, Wayne State University School of Medicine, Detroit, MI, United States; ^3^Department of Psychiatry and Psychotherapy, Medical University of Vienna, Vienna, Austria; ^4^Division of Nuclear Medicine, Department of Biomedical Imaging and Image-guided Therapy, Medical University of Vienna, Vienna, Austria; ^5^Department of Neurology, Medical University of Vienna, Vienna, Austria; ^6^Department of Pediatrics, Wayne State University School of Medicine, Detroit, MI, United States

**Keywords:** resting-state fMRI, Cerebral metabolic rate of glucose, integrated PET/MRI, glucose metabolic rate variability, standardization of psychological state, real-time fMRI

## Abstract

In the past, determination of absolute values of cerebral metabolic rate of glucose (CMRGlc) in clinical routine was rarely carried out due to the invasive nature of arterial sampling. With the advent of combined PET/MR imaging technology, CMRGlc values can be obtained non-invasively, thereby providing the opportunity to take advantage of fully quantitative data in clinical routine. However, CMRGlc values display high physiological variability, presumably due to fluctuations in the intrinsic activity of the brain at rest. To reduce CMRGlc variability associated with these fluctuations, the objective of this study was to determine whether functional connectivity measures derived from resting-state fMRI (rs-fMRI) could be used to correct for these fluctuations in intrinsic brain activity. Methods: We studied 10 healthy volunteers who underwent a test-retest dynamic [18F]FDG-PET study using a fully integrated PET/MR system (Siemens Biograph mMR). To validate the non-invasive derivation of an image-derived input function based on combined analysis of PET and MR data, arterial blood samples were obtained. Using the arterial input function (AIF), parametric images representing CMRGlc were determined using the Patlak graphical approach. Both directed functional connectivity (dFC) and undirected functional connectivity (uFC) were determined between nodes in six major networks (Default mode network, Salience, L/R Executive, Attention, and Sensory-motor network) using either a bivariate-correlation (R coefficient) or a Multi-Variate AutoRegressive (MVAR) model. In addition, the performance of a regional connectivity measure, the fractional amplitude of low frequency fluctuations (fALFF), was also investigated. Results: The average intrasubject variability for CMRGlc values between test and retest was determined as (14 ±8%) with an average inter-subject variability of 25% at test and 15% at retest. The average CMRGlc value (umol/100 g/min) across all networks was 39 ±10 at test and increased slightly to 43 ±6 at retest. The R, MVAR and fALFF coefficients showed relatively large test-retest variability in comparison to the inter-subjects variability, resulting in poor reliability (intraclass correlation in the range of 0.11–0.65). More importantly, no significant relationship was found between the R coefficients (for uFC), MVAR coefficients (for dFC) or fALFF and corresponding CMRGlc values for any of the six major networks. Discussion: Measurement of functional connectivity within established brain networks did not provide a means to decrease the inter- or intrasubject variability of CMRGlc values. As such, our results indicate that connectivity measured derived from rs-fMRI acquired contemporaneously with PET imaging are not suited for correction of CMRGlc variability associated with intrinsic fluctuations of resting-state brain activity. Thus, given the observed substantial inter- and intrasubject variability of CMRGlc values, the relevance of absolute quantification for clinical routine is presently uncertain.

## Introduction

Pioneering studies in the early days of PET imaging have demonstrated the potential of absolute quantification of glucose metabolic rate using [18F]-labeled deoxy-glucose (FDG) (Lammertsma, 2017). Absolute quantification studies typically mandate lengthy dynamic imaging protocols, along with the measurement of arterial blood samples (Schmidt and Turkheimer, 2002). Due to the complexity associated with the imaging protocols and surprisingly large inter- and intra-subject variability (in the range of 15–25%) of the cerebral metabolic rate of glucose (CMRGlc) (Chang et al., 1987; Tyler et al., 1988; Camargo et al., 1992), absolute quantification studies have not been clinically viable.

Under normal physiological conditions, the brain derives most of its energy from glucose metabolism (Raichle and Mintun, 2006). The observed substantial variability of glucose metabolic rate was mostly unexpected, especially as it was shown that changes in moment-to-moment energy demands may contribute as little as 0.5–1% to the total energy budget (Raichle and Mintun, 2006). This implies that intrinsic brain activity may be an important factor in terms of overall brain function. It is believed that this intrinsic activity is an expression of recurrent excitatory and inhibitory connections between and within layers of the cerebral cortex that are fundamental to the operation of local cortical circuits (Haider et al., 2006).

Currently the underlying factors that give rise to the observed variability in intrinsic brain activity are unknown. However, it has been speculated that it might be an expression of fluctuations in vigilance and conscious awareness, which is strongly tied to electrical and chemical signaling at neuronal synapses (Fukunaga et al., 2008). Thus, the brain’s intrinsic energy consumption, as measured using FDG-PET imaging might be closely linked to the function of neural regulatory networks that underlie affective and cognitive processes. These processes are known to be associated with the hemodynamic response that can be studied based on spontaneous oscillations captured during resting-state fMRI (rs-fMRI) (Logothetis et al., 2001). With the advent of fully integrated PET/MR imaging (PET/MRI), both CMRGlc, as well as rs-fMRI can be acquired contemporaneously, paving the way for the study of the relationship between moment-to-moment blood flow changes and longer-lasting states of brain energy consumption. Here, we investigate whether a relationship exists between CMRGlc values and the functional connectivity between major nodes within the larger brain network. Such a relationship would allow standardization of CMRGlc values to a subject’s resting-state intrinsic activity, which may provide a means to decrease test-retest variability of CMRGlc values and may improve the relevance of absolute quantification of glucose metabolic rates in clinical routine.

## Materials and Methods

### Subjects

Ten healthy volunteers (27 ± 7 years, 5M/5F) were included in this study. The study was approved by the Ethics Committee of the Medical University of Vienna (EK1960/2014) and was performed in accordance with the Gandavia and Tovella (1964), including current revisions. Volunteers were deemed to be healthy based on their medical history, physical examinations, and vital signs. Written informed consent was obtained from all the subjects before the examinations.

### PET/MR Imaging Protocol

This study was part of an ongoing project with the goal to validate methodology that allows the determination of an accurate image-derived arterial input function (AIF) (Sundar et al., 2019). All volunteers underwent test-retest examinations (mean time difference = 17 ± 44 days) in a fully integrated PET/MR system (Siemens Healthineers Biograph mMR, Erlangen, Germany). Examinations were conducted in the afternoon and subjects were asked to keep their eyes open and to relax without thinking of anything in particular.

Subjects were fasted for at least 6 h prior to the PET imaging procedure. Before each scan, blood glucose levels (mmol/l) were measured and a venous line was established for the injection of the FDG tracer. In addition, an arterial line was established in the contra-lateral arm of the subjects for manual arterial blood sampling. After positioning the subject in the PET/MR system with the brain in the field-of-view (FOV), a 60-min PET list-mode acquisition was initiated simultaneously with an intravenous injection of (352 ± 66 MBq, 5.2 MBq/kg) FDG administered manually as a slow bolus over 40 s.

Parallel to the PET data acquisition, multiple MR sequences were acquired: a T1-w MR sequence (TR: 2200 ms, TI: 778 ms, TE: 3 ms, flip-angle = 13°, FOV: 256 mm × 256 mm, 256 axial slices of thickness = 1.0 mm, matrix = 256 × 256, scan-time = 5 min 22 s) for anatomical localization as well as a rs-fMRI sequence (TR: 2.44 s, TE: 29 ms, FOV: 256 mm × 256 mm, acquisition matrix: 128 × 128, 36 axial slices, voxel dimensions: 2 mm × 2 mm × 3 mm, 170 images for a total of 7 min). The fMRI sequence was initiated at 30 min post injection of the FDG tracer to match the linear component of the Patlak-transformed time-activity curves (see below). In addition, sparsely sampled MR navigators (2D EPI 3.0 mm × 3.0 mm × 3.0 mm voxels, 64 × 64 matrix, 36 slices, TE = 30 ms, TR = 3000 ms) were interleaved between clinical MR sequences with the following time intervals: 0, 2.5, 5, 7.5, 10, 14, 17, 21, 26, 33, 38, 42, 44, and 50.5 min post-injection.

Following the PET/MR examination, the controls were moved to the PET/CT for a low-dose CT scan (120 kVp, 50 mAs) of the brain. The PET list-mode data was re-binned into a dynamic frame sequence (24 × 5 s, 1 × 60 s, 1 × 120 s, 11 × 300 s) and was reconstructed (Siemens e7 tools) into a 344 × 344 × 127 matrix (voxel size 2.08 mm × 2.08 mm × 2.03 mm) using the ordinary Poisson ordered subset expectation-maximization (OP-OSEM) 3D algorithm (3 iterations, 21 subsets, 2 mm Gaussian filter). Brain attenuation correction was performed using a CT-derived mu-map (Carney et al., 2006), which was co-registered to the navigator image volumes, yielding dynamic AC-maps along with scatter correction.

### Blood Sampling

To obtain the AIF, blood samples were collected manually at different time points (24 × 5 s, 1 × 60 s, 1 × 120 s, 1 × 300 s, 1 × 600 s, 2 × 1200 s post injection) from the radial artery. The blood sampling was performed manually using vacuum test tubes via an arterial cannula fitted with an adapter. Before every arterial sample, the line was flushed with 5 mL sodium chloride solution to prevent clotting and sampling stagnant blood. To avoid dilution of the actual sample, a 1 mL of discard was drawn followed by the sampling of the arterial blood sample. Whole-blood radioactivity concentrations were measured using a gamma counter (PerkinElmer, 2480 Automatic Gamma counter, Wizard23). To obtain the AIF, whole blood samples were centrifuged to separate the plasma component, followed by the measurement of radioactivity in the plasma.

### MR-Driven Motion Correction

Sparsely sampled MR navigators interleaved between MR sequences were used to perform motion correction of PET images (Keller et al., 2015). The initial navigator (Nav-0) was considered as the reference volume, and all subsequent navigators (Nav-1 to Nav-13) were rigidly aligned to Nav-0 using SPM 12 (Wellcome Trust Center for Neuroimaging, UCL), yielding a set of motion vectors (MV-1 to MV-13, three translations, and three rotation parameters). A correspondence between the MR navigators and PET emission data was assumed based on the least temporal difference between the MR navigator acquisition time and the PET frame mid-scan time. To account for spatial misalignment between the static CT-derived AC map and the PET emission data, the inverse of the MVs (iMVs) were applied to the AC map, which resulted in a set of motion-corrected AC maps (MoCo-AC). The obtained MoCo-AC maps were then employed for reconstruction of the dynamic PET emission data using the Siemens e7 tools.

### PET Quantification

Motion vectors (MVs) derived from the MR navigators were applied to the corresponding PET frames, resulting in motion-corrected PET frames (MoCo-PET). Following the spatial alignment, a voxel-wise Patlak graphical analysis (lumped constant, LC = 0.65) (Wu et al., 2003) was performed using time-activity curves derived from MoCo-PET frames in combination with the sampled AIF. The analysis was performed using an in-house developed Matlab tool (Matlab R2018a, MathWorks, United States) that generated parametric images representing the CMRGlc in units of umol/100 g/min. To be specific, a linear function was fitted to the Patlak-transformed data, including data from 25 min p.i. until the end of the study (8 data points). The resulting slope was then multiplied with the subject’s plasma glucose level (umol/L) and divided by the LC (Supplementary Figure S2).

### fMRI Preprocessing

The fMRI images were analyzed using SPM12 (Wellcome Department of Cognitive Neurology, Institute of Neurology, London, United Kingdom). In all analyses, the first four images were discarded to account for echo planar imaging (EPI) equilibration effects (Haacke, 2014). The remaining images in the sequence were realigned to correct for head movements, corrected for slice timing, and subsequently spatially normalized based on the transformation matrix derived between the co-registered (to the mean EPI image) T1-weighted image and the MNI template brain. The images were then smoothed spatially with a 3D Gaussian kernel of 6 mm FWHM and re-sampled (2 mm × 2 mm × 2 mm).

### fMRI Time Series Extraction

**FIGURE 1:**
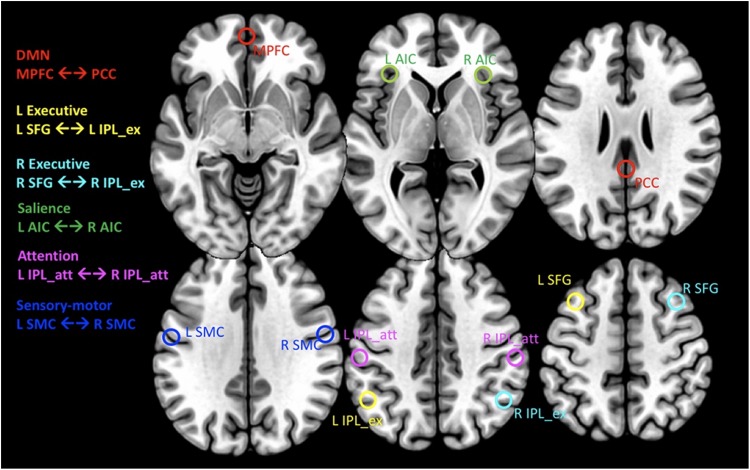
Transaxial planes showing the location of spherical regions in MNI space from where the CMRGlc values and rs-fMRI time series’ were extracted. For MNI coordinates of regions, see text. Red: default mode network (DMN) connecting the medial prefrontal cortex (MPFC) with the posterior cingulate cortex (PCC). Yellow and Cyan: L/R Executive networks, connecting the L/R superior frontal gyrus (SFG) with the anterior portion of the inferior parietal lobe (IPL_ex), respectively. Green: salience network connecting the R/L anterior insular cortex (AIC). Magenta: attention network connecting the L and R posterior portion of the inferior parietal lobe (IPL_att). Blue: sensory-motor cortex connecting the L and R sensory motor cortex (SMC).

A subset of six pair-wise nodes was selected for our analysis [see Supplementary Figure S3 in Biswal et al. (2010)]. These nodes are defined by their peak coordinates and correspond to the following major networks: the default mode network (DMN) including the medial prefrontal cortex (MPFC, MNI coordinates [0/60/−6]) and the posterior cingulate cortex (PCC, MNI [3/−42/27]), the R/L executive network connecting the right/left superior frontal gyrus (SFG, MNI [±30/21/51]) with the right/left inferior parietal lobe (IPL, MNI [±48/−57/42]) within each hemisphere, the salience network connecting the L and R anterior insula cortex (AIC, MNI [−36/18/3] ↔ [42/15/−3]), the attention network connecting the L and R IPL (MNI [−54/−30/42] ↔ [42/−36/48]) and the sensory-motor cortex connecting the L and R superior central sulcus (MNI [−57/−9/33] ↔ [60/−9/33]). For each peak location, the time series of all voxels within a radius of 6 mm were averaged. These averaged time-series were then used to determine both the directed and undirected functional connectivity (FC) between pair-wise nodes constituting the selected networks (Figure 1). Specifically, the directed functional connectivity (dFC) model is well suited for assessing (possibly) asymmetrically directed interactions between any nodes in any class of network with quantifiable dynamics (Granger, 1969; Bressler and Seth, 2011; Friston, 2011). Moreover, in order to assess local functional connectivity (which characterizes the extent of temporal coherence between neighboring voxels) we also calculated the fractional amplitude of low frequency fluctuations (fALFF). This measure of local connectivity has been shown to be associated with brain activity (Yu-Feng et al., 2007; Zhou et al., 2010).

### Undirected Functional Connectivity Analysis

Undirected functional connectivity (uFC) analysis was performed using previously published methods (Whitfield-Gabrieli and Nieto-Castanon, 2012). Segmentation of structural (T1-w) images was performed and the resulting gray matter and cerebrospinal fluid (CSF) images were co-registered with the functional (T2-w) scans. The BOLD signals from white matter and CSF masks as well as the motion regressors were set as confounds, using the default orthogonal time series. The temporal confounding factors were then regressed from the BOLD time series at each voxel and the residual time series’ were band-pass filtered (0.01–0.1 Hz) to eliminate low frequency drifts. ROI time series’ were extracted by averaging across all the voxels within each individual ROI. Finally, uFC between two regions was computed using a zero-lagged bivariate-correlation *R*-value), estimating the linear association between two BOLD signals.

### Directed Functional Connectivity Analysis

Directed Functional Connectivity was estimated using Multi-Variate AutoRegressive (MVAR) models. These models provide a measure of the causal influence of each anatomical node on every other node in the network and are equivalent to Granger Causality (GC) (Granger, 1969). In brief, GC relies on the notion of “prediction” to generate influences regarding “causality.” A constituent “X” within a complex dynamic system exerts a “causal” effect on another constituent “Y” within the system if the predictability of “Y” decreases when “X” is removed from the set of all possible causative variables. The most typically used framework relies on auto-regressive models (Bressler and Seth, 2011). Thus, the MVAR model was used to estimate the strength of the causal influence between nodal pairs (A, B: A → B, B → A), with the model coefficient encoding the magnitude of this strength (Bressler and Seth, 2011; Tang et al., 2012; Asemi et al., 2015; Diwadkar et al., 2017; Morris et al., 2019). The number of previous time points in the model that was used to estimate the current time point was restricted to one (Tang et al., 2012), since network interactions at time scales that are proximate to the cognitive neuro-dynamics of the brain networks are in the time range of milliseconds (Singh, 2012).

All modeling was performed using specifically written scripts (R software suite). For each nodal pair (A, B) two MVAR coefficients were estimated for each subject characterizing the direction of the pair (A → B and B → A). Thus, each participant contributed 24 coefficients to the group level analyses (6 pairs from 12 nodes; two directions for each pair over the test and retest conditions).

### Fractional Amplitude of Low Frequency Fluctuations Analysis

Fractional amplitude of low frequency fluctuations (Zou et al., 2008) is defined as the power within the low-frequency range (0.01–0.08 Hz) divided by the total power in the entire detectable frequency range and is calculated for the time course of each voxel within a ROI. These values were averaged to yield one regional value for the 10 nodes. The rationale for using this measure is based on the assumption that slow fluctuations in activity are a fundamental feature of the resting brain, and their presence is crucial for determining correlated activity between brain regions that constitute resting state networks.

### Multimodal Analysis

In order to determine the relationship between CMRGlc and functional connectivity of major brain networks (Figure 1), CMRGlc values were derived from the same coordinate locations as were used for the fMRI data analysis. Specifically, parametric images representing CMRGlc were co-registered with the corresponding T1-w images and following spatial normalization of the T1-w images to MNI space using DARTEL (Diffeomorphic Anatomic Registration Through Exponentiated Lie algebra) software, these normalization parameters were also applied to parametric CMRGlc images. Thereafter, CMRGlc values were extracted from spheres with a 6 mm radius centered at the same location as was used for the extraction of the fMRI time series. Next, CMRGlc values obtained from the 12 nodes were averaged separately for each network, yielding for each subject six network-specific regional CMRlc values for both the test (rCMRGlc_*tst*_) and retest (rCMRGlc_*retst*_) condition. A correlation analysis was then performed in order to determine whether the magnitude of the uFC (expressed by Pearson’s R coefficients), dFC (expressed by the MVAR coefficients) of local connectivity (characterized by fALFF) is predictive of CMRGlc values at rest and retest conditions. In case of a significant correlation between CMRGlc values and the R/MVAR/fALFF coefficients (COEF), these coefficients could be applied to account for CMRGlc variability by adjusting the retest CMRGlc values to the psychological state during the test condition as

(1) CMRGlc=′retestCMRGlcretest×[COEFtest/COEFretest]

where CMRGlc’_*retest*_ represents the adjusted glucose metabolic rate.

### Statistical Analysis

Descriptive statistics was used to characterize the average and the variance of outcome measures (CMRGlc values, R, MVAR, and fALFF coefficients) determined at test and retest condition for the six major networks. Moreover, the test-retest repeatability of each outcome measure was assessed using the two-way random effects model intraclass correlation coefficient [ICC (2,1)]. To determine whether there is a significant difference with respect to outcome measures across time and network, a (2 × 6) repeated measures ANOVA was applied, where the two within-subjects factors represent time with 2 levels (test, retest) and networks with 6 levels (DMN, Salience, L/R Executive, Attention, Sensory-motor). All tests were performed 2-sided and a *p*-value of <0.05 was assumed to represent significance. Pearson’s correlation was used in order to assess whether a significant correlation exists between CMRGlc values and the R or MVAR coefficients. For correlation analyses, individual values were transformed to z-scores prior to correlation computation. Statistical analysis was performed using SPSS version 25 (SPSS, Inc.).

## Results

**FIGURE 2:**
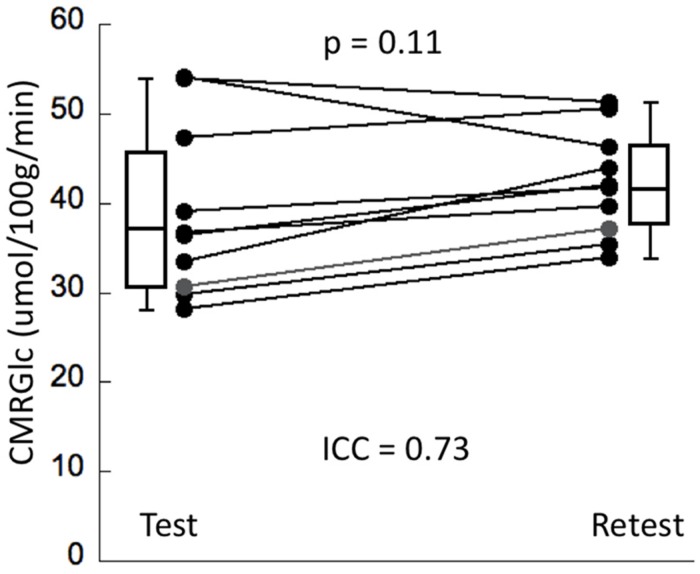
The panel displays whole brain CMRGlc values at test and retest condition (*N* = 10). Although the average CMRGlc value across the two conditions is similar, the variability is decreased during the retest condition (see Table 1).

Cerebral metabolic rate of glucose values at rest and retest condition obtained for the six major networks are shown in Table 1. The average intrasubject variability between rest and retest was determined as (14 ±8%) with an average inter-subject variability of 25.4% at rest and 15.1% at retest, representing a trend toward a significant decrease in variability during retest (*F* = 2.7, *p* = 0.08). The intraclass correlation coefficient (ICC) for CMRGlc values determined in the six networks was in the range of 0.68–0.78, indicating a moderate agreement between the two time points. The average CMRGlc value over all networks was (39 ±10) umol/100 g/min at test and increased slightly to (43 ±6) umol/100 g/min at retest (*p* = 0.11) (Figure 2). As expected, the repeated measures ANOVA showed a highly significant main effect for the network variable (*p* < 0.001), indicating significant differences of CMRGlc values in the individual networks. Despite the significant differences in absolute CMRGlc values between test and retest condition, the ratio between CMRGlc values in individual regions was preserved across the two time points (as verified by a non-significant (time × network) interaction of *p* = 0.47).

**TABLE 1:** CMRGlc values determined for the test (Test) and retest (Retest) condition in each of the six major brain networks.

**Network**	**Test (COV) CMRGlc (umol/100 g/min)**	**Retest (COV) CMRGlc (umol/100 g/min)**	**Intrasubject variability**
DMN	41 ± 10 (24)	45 ± 6 (12)	14 ± 8
L executive	41 ± 11 (24)	44 ± 8 (17)	14 ± 8
R executive	40 ± 10 (26)	42 ± 7 (16)	13 ± 7
Salience	41 ± 10 (25)	45 ± 6 (13)	14 ± 9
Attention	36 ± 9 (26)	39 ± 6 (12)	15 ± 8
Sensory-motor	35 ± 8 (24)	37 ± 6 (12)	15 ± 8
Average	39 ± 10 (25)	43 ± 6 (15)	14 ± 8

*The coefficient of variation (COV = mean/SD) is provided in brackets. “Average” represents the overall mean derived from all nodes across all subjects.*

### Reliability of Functional Connectivity Measures

**FIGURE 3:**
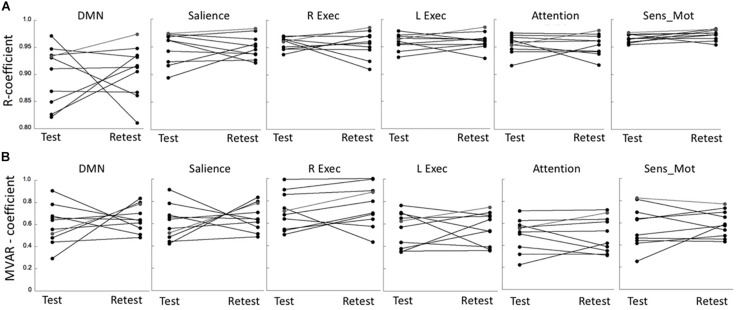
**(A)** Test-retest values of R coefficients in six major networks: Default Mode network (DMN), Salience network (Salience), R/L Executive network (R Exec/L Exec) and Attention network (Attention) and Sensory-motor network (Sens_Mot). **(B)** Corresponding test-retest values of MVAR coefficients in the same six major networks. R, bivariate-correlation coefficient; MVAR, Multi-Variate Auto-Regressive model coefficient.

The R coefficients at test and retest conditions for all six networks are shown in Figure 3A, indicating a relatively large test-retest variability in comparison to the between-subjects variability. This was reflected in the poor network ICC values that were determined in the range of 0.11–0.36. Nevertheless, the repeated measures ANOVA showed a non-significant effect both with respect to the main effect for time (*p* = 0.95) as well as for the (time × network) interaction (*p* = 0.89). A similar result was determined for the MVAR coefficients (Figure 3B). The network ICC values were determined in the range 0.14–0.55, with the repeated measures ANOVA again showing a non-significant time effect (*p* = 0.71) and (time × network) interaction (*p* = 0.67). For both analyses, the highest overall connectivity was determined for the Sensory-motor network (*R* = 0.97 and MVAR = 0.59 for uFC and dFC, respectively) while the DMN displayed lowest connectivity (*R* = 0.88 and MVAR = 0.41). Finally, the network ICC values for the fALFF parameter were determined in the range of 0.33–0.65 with a non-significant time (*p* = 0.92) and (time × region) interaction effect (*p* = 0.87) based on a repeated measures ANOVA.

### Relationship Between CMRGlc and Functional Connectivity

**FIGURE 4:**
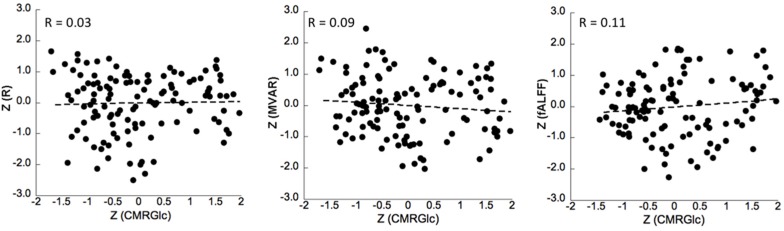
Correlation between z-transformed CMRGlc values and z-transformed R (left), MVAR (middle) and fALFF (right) coefficients for all subjects in six networks at test and retest conditions. The *R*-value represents the Pearson’s correlation coefficient between the measures. R, bivariate-correlation coefficient; MVAR, Multi-Variate Auto-Regressive model coefficient; fALFF, fractional amplitude of low frequency fluctuations.

No significant correlation was found between corresponding CMRGlc values and either of the R, MVAR or fALFF coefficients for any of the six major networks. Correlation analysis showed a very poor correlation between CMRGlc values and R coefficients with an r^2^ value of <0.02 for all individual networks. A similar result was determined for the MVAR coefficients characterizing dFC for all networks and directions as well as for the fALFF parameter. Figure 4 shows representative correlation graphs between CMRGlc values and the R, MVAR and fALFF coefficients collapsed over all six networks, demonstrating the absence of any meaningful correlation between functional connectivity measures and regional CMRGlc. Accordingly, application of (Eq. 1) did not improve the reproducibility of CMRGlc values between test and retest conditions. The variability of adjusted CMRGlc values was either similar (13 ±6% vs 13 ±8%, sensory-motor cortex) or was significantly worse (24 ±18% vs 16 ±10%, DMN network) as compared to the measured CMRGlc values.

**FIGURE 5:**
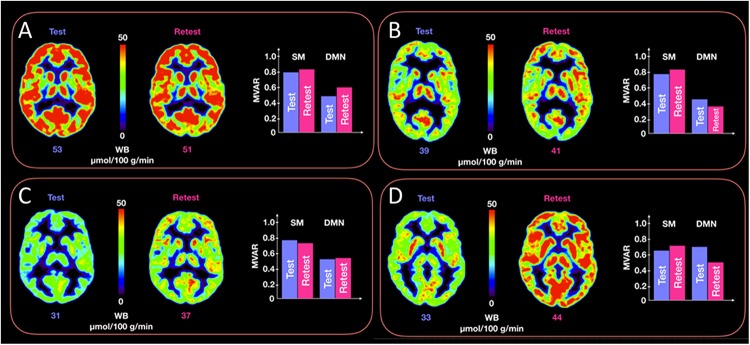
Representative CMRGlc images with low (top row, panels **A,B**) and high (bottom row, panels **C,D**) test-retest variability. Each of the four panels **(A–D)** corresponds to a different subject at rest and retest condition. Each panel renders a trans-axial cross-section through the subject’s brain at the level of the caudate head obtained at rest and retest condition, together with bar graphs representing test-retest changes in MVAR coefficients for the DMN and Sensory-motor (SM) networks. The figure demonstrates a similar distribution of MVAR coefficient changes for subjects with low (top row) and high (bottom row) CMRGlc variability across time. WB, whole brain values.

Figure 5 provides representative CMRGlc images of two subjects with good reproducibility across time (<5% difference in whole brain CMRGlc values) and two subjects with poor reproducibility (>15% difference) together with the corresponding MVAR coefficients for the three selected networks. Although the changes in absolute CMRGlc values differ between the two groups, changes in functional connectivity (as characterized by the MVAR coefficient) between test and retest condition are similar, as indicated in the figure for the DMN and Sensory-motor network. The poor agreement between temporal changes in whole brain CMRGlc values and changes in the MVAR coefficient in two representative brain networks can be clearly appreciated.

## Discussion

The main result of our study is the absence of a relevant relationship between absolute metabolic rate of glucose values and measures of functional connectivity in six major brain networks (Figure 4). Our results indicate that functional connectivity between major network nodes (as quantified using R, MVAR or fALFF coefficients derived from rs-fMRI) is not suited for standardization of CMRGlc values with respect to the subject’s intrinsic network activity (Figure 5). Thus, given the substantial intra- and intrasubject variability of glucose metabolic rate, the usefulness of absolute quantification in clinical routine remains to be determined.

The current study confirms the poor test-retest reliability of functional connectivity measures reported in recent literature (Noble et al., 2019). These investigators performed a meta-analytic estimate of the reliability of fMRI based functional connectivity in 44 studies, stating an overall poor ICC of 0.29 (95% CI = 0.23–0.36). One potential reason for the observed low ICC of functional connectivity measures is the relatively large intra-subject variability compared to a comparatively low inter-subject variability (see Figure 3).

Moreover, our results both complement and extend the findings of a recent study (Parker and Razlighi, 2019) that demonstrates that consistent and robust deactivations in task-based fMRI studies can be significantly altered without causing any changes in their overlapping intrinsic functional connectivity. These authors present compelling evidence for a disassociation between task-evoked deactivations and functional connectivity, both extracted from the same DMN regions. Moreover, they demonstrate that task-based deactivations are more closely related to task performance than is functional connectivity. Based on this data the authors conclude that functional connectivity represents a distinct and ongoing neuro-physiological process whose coherence and magnitude is not altered by task-performance, but is taking on a more basic role in the hierarchical functional architecture of the brain.

Recent studies have demonstrated that task-related changes in brain activity levels are associated with changes in glucose consumption (Riedl et al., 2014; Jamadar et al., 2019), presumably due to altered energy demand of the underlying synaptic transmission processes. Using simultaneous acquisition of both FDG-PET and fMRI data, these investigators have shown a close relationship between task-based functional connectivity and local glucose metabolism in the visual network, consistent with results obtained in non-simultaneously acquired data (Di and Biswal, 2012; Tomasi et al., 2017). Thus, as CMRGlc is closely tied to task-performance, our results showing a poor correlation between glucose metabolic rate and non-task based functional connectivity, directly corroborate Parker and Razlighi (2019) interpretation. Finally, our results are also in partial agreement with a study by Marchitelli et al. (2018), who set out to investigate the coupling between FDG tracer uptake and intrinsic functional activity in both patients (Alzheimer Disease) and a control group. These investigators showed only a modest across-subjects correlation between FDG tracer uptake and intrinsic functional activity, indicating that the variability observed with functional connectivity measures is at least as large as the variability of glucose consumption. Thus, both studies indicate that functional connectivity measures cannot provide added information that could be used to account for the observed physiological variability in glucose metabolic rate.

### Physiological Variability of CMRGlc Values

Fully quantitative assessment of CMRGlc provides valuable and detailed information about the regional metabolic state of brain tissue, but this advance in methodology brings its own set of issues that need to be carefully considered. Early studies that have investigated absolute CMRGlc in control subjects have revealed a surprisingly large physiological variability that was in the range of 15–25%, even for large regions and the same subject being scanned only a few days apart (Camargo et al., 1992; Schaefer et al., 2000). Our own data confirms these earlier findings (Figure 2). Consequently, in the absence of improved data acquisition or analysis protocols that are able to standardize the resting-state metabolism of the subjects, sensitivity to detect areas of significantly increased or decreased CMRGlc will be relatively low, requiring about 30% deviations from baseline (Sundar et al., 2019). This compares unfavorably with the visual assessment of regional asymmetries between homotopic brain areas, which can be quite easily detected at the 10% level (Niimura et al., 1999). Thus, in order to improve the relevance of absolute quantification in clinical applications, standardization of the subjects’ resting-state activity will be mandatory. Unfortunately, it is currently unclear how such a standardization could be achieved.

Our previous work showed that the observed physiological variability does not correlate with the time duration between the two PET scans (Sundar et al., 2019), suggesting that variability is not due to a slow drift of brain metabolism across time, but appears to be an inherent characteristic of the underlying neural network. This effect has been extensively studied in the context of the DMN, originally proposed by Raichle et al. (Gusnard et al., 2001; Raichle and Snyder, 2007). The DMN displays fluctuation of brain activity during times when a subject is not performing any task but is left in an “idle” state. These fluctuations are believed to be the result of unconstrained, spontaneous cognition - daydreams or, more technically, stimulus-independent thoughts (McGuire et al., 1996; Mason et al., 2007), which recently led to the realization that a truly “resting” state of the brain probably does not exist (Gusnard et al., 2001). Moreover, such a state is in general undefined as various neural processes that are currently uncontrollable are likely to contribute to the observed variability in CMRGlc. As a case in point, our data suggests that higher-order brain networks (such as the DMN and R/L executive networks) show higher temporal variability than does the sensory-motor network. A possible reason for this surprising finding might be the fact that intrinsic flexibility in these high-order brain networks in combination with complex between-network interactions (Calhoun et al., 2014) may increase their sensitivity to transient environmental factors that play an important role in the mental state of a patient. In contrast, the functional connectivity within the sensory-motor system is relatively independent of transient factors and as a result, might more stably reflect the baseline state of the brain.

### Temporal Relationship Between CMRGlc and rs-fMRI

In order to calculate CMRGlc values, dynamic PET data over an extended time period (∼60 min) is acquired. Moreover, to derive CMRGlc values from such data, a three-compartment model (consisting of a vascular, intracellular and metabolic compartment) is applied. Analysis of such a compartmental model indicates that after ∼25 min post injection a dynamic equilibrium is reached between the compartments which, under resting conditions, is maintained until the end of the study (∼60 min). The presence of such a dynamic equilibrium suggests that during this time the CMRGlc is unchanged and can be therefore uniquely identified from the data. This assumption is well supported by the observation that the Patlak-transformed dynamic time-activity curves display a linear behavior during the dynamic equilibrium phase, allowing a simplified analysis based on linear fitting. Thus, it appears reasonable to assume that during dynamic equilibrium the functional EPI data should also reflect a stable network configuration commensurate with the measured CMRGlc values (see Supplementary Figure S1 in the Supplementary Material).

### Neurovascular Coupling

It is reasonable to assume that resting-state glucose is physiologically related to baseline neural activity as well as to resting cerebral blood flow (Attwell and Iadecola, 2002). Although rs-fMRI is considered to represent an indirect measure of neural activity in specified neural networks, several issues prevent a straightforward application of rs-fMRI derived parameters to yield information about overall energy consumption in these networks.

Firstly, the mechanisms underlying neurovascular coupling and neuronal function are still not completely understood (Leithner and Royl, 2014). For example, it is not fully understood what function moment-to-moment changes in blood flow serve. The delivery of oxygen and glucose are indeed an important factor, but the availability of reserves (i.e., unextracted oxygen in circulating blood and glucose and glycogen in astrocytes) make a simple relationship unlikely. Possible other mechanisms that have been proposed are the removal of excess lactate produced during an increase in activity or the adjustment of the acid-base/ionic balance of the tissue. However, these mechanisms do not lend themselves easily to a transparent relationship with functional connectivity.

Secondly, established measures of local connectivity such as Regional Homogeneity (ReHo, Zang et al., 2004) and fractional Amplitude of Low Frequency Fluctuations (fALFF, Zou et al., 2008) represent local variables closely related to small (<3%) moment-to-moment regional blood flow changes, but not to absolute blood flow values (which can be non-invasively measured using 15O-water PET imaging). Both these parameters have been shown to correlate with local FDG uptake (Tomasi et al., 2013; Aiello et al., 2015; Savio et al., 2017; Rajkumar et al., 2018), but a relationship with absolute glucose metabolic rates could not be demonstrated.

Finally, Zuo et al. (2012) defined a direct connectivity (DC) parameter which provides a measure of information flow within a network. DC is a local measure of functional connectivity that indexes the number of direct connections for a given node and is calculated as the Pearson’s correlation coefficient between remote voxels (Buckner et al., 2009). Conceptually, a node has high DC if it has numerous direct connections to other nodes and such a node would be expected to be more active than a node with low DC. Surprisingly, DC has been shown to correlate poorly with FDG tracer uptake (Aiello et al., 2015), corroborating our results. Our findings suggest that fluctuations in blood flow measured with rs-fMRI are superimposed on a much larger blood flow baseline that cannot be assessed using fMRI measures. However, it is this (unknown) blood flow baseline, that determines overall energy consumption in the brain (Sokoloff, 1980).

### PET/MR: The Sum Is Greater Than Its Parts

In accordance with the finding that task-based deactivations are more closely related to task performance than is functional connectivity (Parker and Razlighi, 2019), it is reasonable to assume that a subject’s psychological state could be influenced by performing a specific task. There is evidence that task-related psychological states in different patients are more similar than during resting state (Duara et al., 1987). These investigators have demonstrated that by involving the subjects in a picture preference test during the FDG uptake period decreases the variability of CMRGlc values by 60–80%. Interestingly, the same task performed in mild to moderately demented patients (with Mini-Mental-State >15) did not result in any appreciable decreases of CMRGlc variability, indicating that subjects need to have a certain level of engagement with the performed task in order to achieve standardization.

Obtaining a measure of task performance might be an excellent parameter that can be used to characterize a particular psychological state. In this context, combined PET/MRI methodology might provide a highly efficient means to monitor task performance by taking advantage of advanced fMRI protocols. For example, a real-time fMRI (rt-fMRI) neuro-feedback protocol could be applied to regulate the psychological state of the subjects under study (Caria et al., 2012; Weiskopf, 2012; Sulzer et al., 2013; Gerin et al., 2016). In fact, numerous studies have established the effectiveness of the rt-fMRI neuro-feedback approach in being able to change the activity of specific brain regions and even to improve emotion regulation through the willful increase of prefrontal control over the amygdala complex (Young et al., 2017; Mehler et al., 2018; Sorger et al., 2018; Watanabe et al., 2018). Thus, although speculative, a protocol that uses an rt-fMRI neurofeedback paradigm during the FDG uptake period could generate a very similar psychological state in the studied subjects, potentially resulting in excellent temporal reproducibility of PET-derived CMRGlc values.

### Study Limitations

There are several possible issues associated with our study that deserve mentioning. Our study includes a relatively low number of participants (*N* = 10) that were, however, imaged in a test/retest design. As such, our data includes 20 measurements, allowing assessment of both between- and within-subjects effects. Moreover, standardization of MRGlc values to psychological state will need to be applied to individual measurements, rendering statistical averages of lesser importance. Another possible limitation is the relatively heterogenous time difference between the test/retest imaging sessions, with a mean time difference between scans being 17 ± 44 days (range 3 days to 2 months). However, our previous analysis (Sundar et al., 2019) showed no significant correlation between test/retest differences in MRGlc values and the length of time separating the two acquisitions (R^2^ = 0.03). These results suggest that time effects are most likely not responsible for the observed variability in the observed MRGlc values. Finally, we observed a relatively large (but not statistically significant) decrease in inter-subject variability from the test to the retest condition (from 25% to 15%). This decrease in CMRGlc variability represents a confound that might be due to increased familiarity of the subjects with the imaging procedure at the second scan, thereby decreasing uncertainty and anxiety levels. It is conceivable that the so generated expectations allowed the subjects to enter a state of mind that was conducive to the task ahead, in essence standardizing their psychological state and resulting in decreased CMRGlc variability. Although speculative, this observation supports our notion that exerting influence over a subject’s psychological state might be effective in decreasing CMRGlc variability.

### Future Directions

In future studies we expect to build upon the results presented herein. In particular, we will seek to combine the current approach with various metrics that focus on local functional connectivity for the purpose of CMRGlc standardization, such as regional homogeneity (ReHo), ALFF as well as a more systematic investigation of the potential of an independent component analysis (ICA) for this purpose. All these measures have been shown to possess excellent test-retest reliability (Zuo and Xing, 2014), leaving open the possibility that a hybrid approach that takes into account both long distance as well as local functional connectivity measures might provide a means to significantly decrease test-retest variability of glucose metabolic rates.

## Conclusion

Although high expectations have been pinned upon the absolute glucose metabolic rates in a clinical setting, these have not been matched by the results of fully quantitative FDG-PET imaging due to the large intra- and intersubject variability. Presumably, this variability appears to be caused by changes in patients’ intrinsic brain activity, of which the underlying mechanisms are currently poorly understood and as a result are being inadequately controlled for. Our attempt to standardize glucose metabolic rates based on functional connectivity measures determined within six major brain networks was unsuccessful, rendering the clinical relevance of absolute quantification of cerebral glucose metabolism uncertain at present.

## Data Availability Statement

All datasets generated for this study are included in the article/Supplementary Material.

## Ethics Statement

The studies involving human participants were reviewed and approved by the Ethics Committee of the Medical University of Vienna (EK1960/2014). The patients/participants provided their written informed consent to participate in this study.

## Author Contributions

All authors made substantial contributions to conception and design, and/or acquisition of data, and/or analysis, and interpretation of data; participated in drafting the article or revising it critically for important intellectual content; and gave final approval of the version to be submitted and any revised version.

## Conflict of Interest

The authors declare that the research was conducted in the absence of any commercial or financial relationships that could be construed as a potential conflict of interest.
