# Supplementary material for: Fully Integrated PET/MR Imaging for the Assessment of the Relationship Between Functional Connectivity and Glucose Metabolic Rate
Source: Front Neurosci. 2020 Mar 25;14:252. doi: 10.3389/fnins.2020.00252 (PMC7111429; doi:10.3389/fnins.2020.00252)

Supplementary Data

1. The Figure show the normalized (to the integral of the blood input function) FDG PET time-activity curves for network nodes. Specifically, individual data obtained during test (black lines) and retest (red lines) conditions is shown for 9 nodes. These nodes include the default mode network (DMN) comprised of the medial prefrontal (MPF) and posterior cingulate cortex (PCC), the L/R executive network comprised of the L/R superior frontal (Sup Front) and inferior parietal (Inf Pariet) cortex, the L/R sensory-motor (Sens Mot) cortex and the R anterior insular cortex (Ant Insula). The time duration of fMRI data acquisition is highlighted in gray. The graph demonstrates an approximately constant curvature of time-activity curves beyond 30 minutes post injection, suggesting a stable tissue glucose metabolic rate during this time interval.

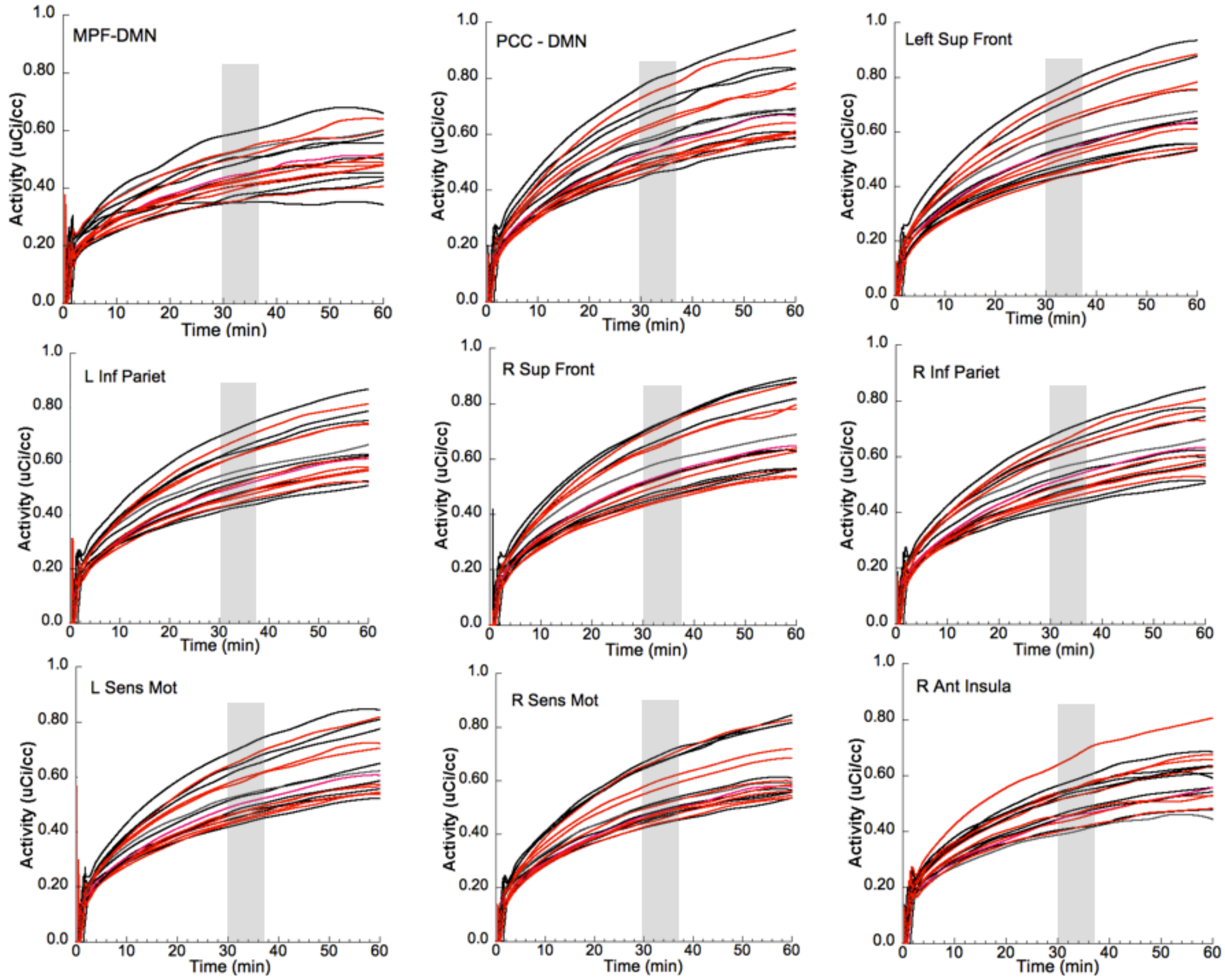

2. The Figure shows the variability of plasma glucose level measurements that were determined in the group of fasted controls (N=10). These values were used to calculate CMRGlc by multiplying these values with the fitted slope of the Patlak-plot (from 25 to 60min post injection) and dividing the result with the lumped constant (LC = 0.65). Although all plasma glucose values were in the normal fasted range (<100 mg/dL), retest values were significantly higher than those at test condition (90 ± 6 vs 95 ± 4 mg/dL; p = 0.01).

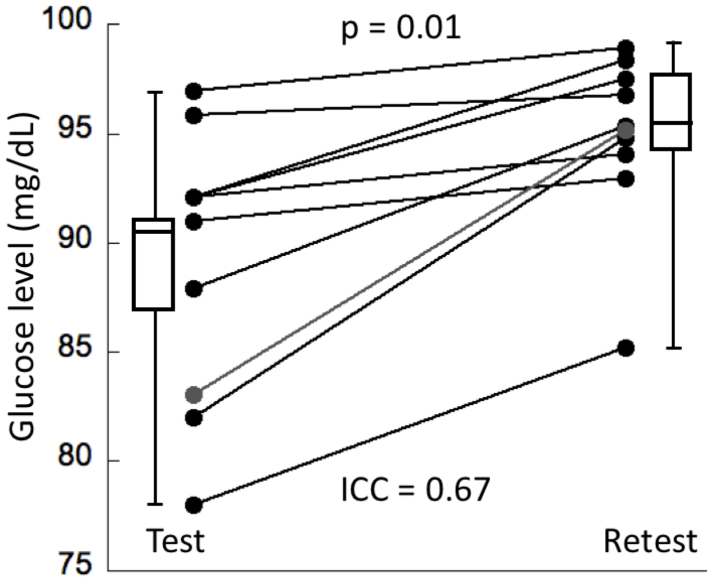

Supplement: Supplementary file 1 [file Data_Sheet_1.PDF]
